# Supplementary material for: Inflammatory factors and risk of lung adenocarcinoma: a Mendelian randomization study mediated by blood metabolites
Source: Front Endocrinol (Lausanne). 2024 Aug 27;15:1446863. doi: 10.3389/fendo.2024.1446863 (PMC11384989; doi:10.3389/fendo.2024.1446863)
Supplement: Supplementary file 1 [file DataSheet1.zip › supplementary files/S2/GCST90200604/sensitivity-analysis.pdf]

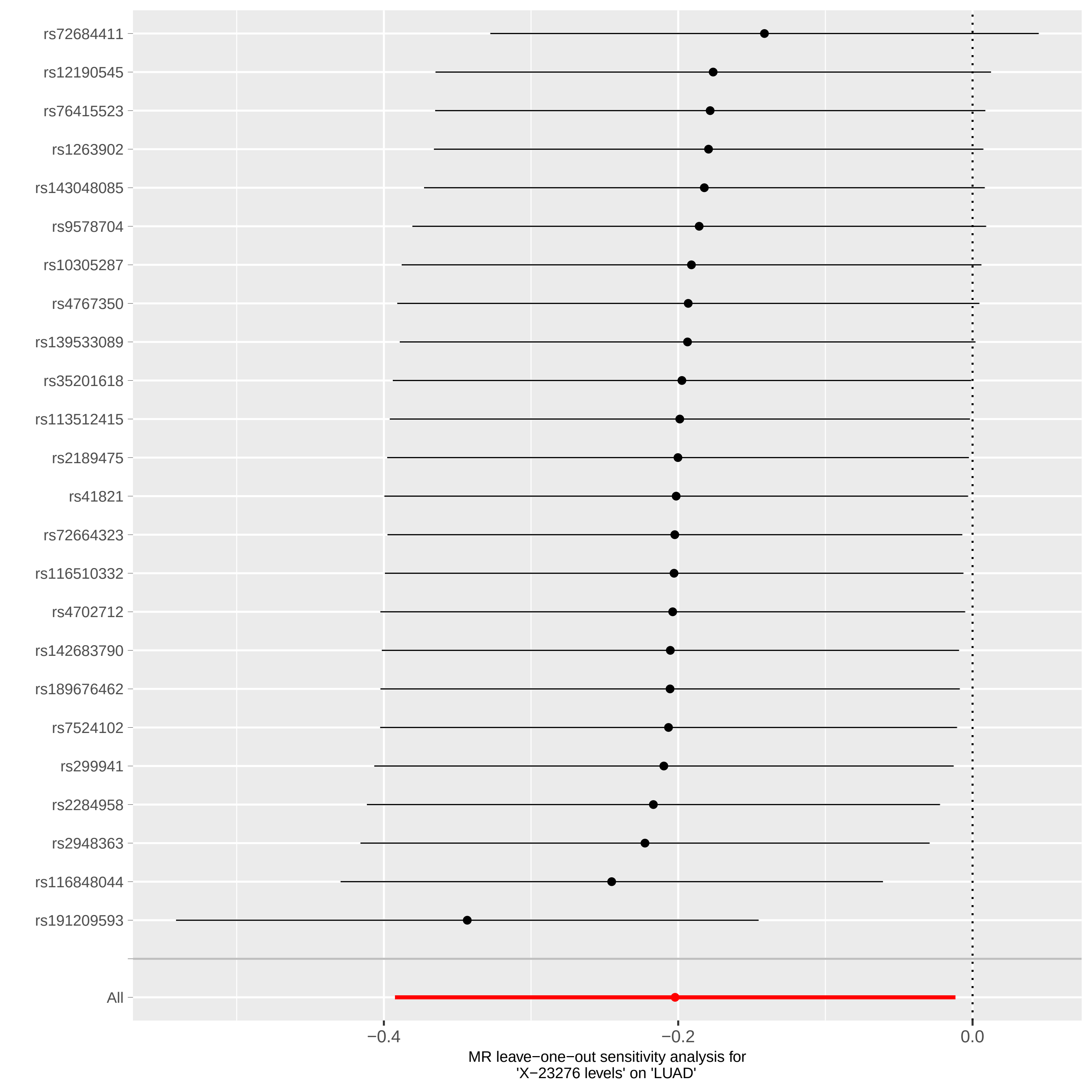
rs72684411
rs12190545
rs76415523
rs1263902
rs143048085
rs9578704
rs10305287
rs4767350
rs139533089
rs35201618
rs113512415
rs2189475
rs41821
rs72664323
rs116510332
rs4702712
rs142683790
rs189676462
rs7524102
rs299941
rs2284958
rs2948363
rs116848044
rs191209593
All
−0.4
−0.2
0.0
MR leave−one−out sensitivity analysis for
'X−23276 levels' on 'LUAD'
